# Genomic Mosaicism of the Brain: Origin, Impact, and Utility

**DOI:** 10.1007/s12264-023-01124-8

**Published:** 2023-10-29

**Authors:** Jared H. Graham, Johannes C. M. Schlachetzki, Xiaoxu Yang, Martin W. Breuss

**Affiliations:** 1 Department of Pediatrics, Section of Clinical Genetics and Metabolism, University of Colorado School of Medicine, Aurora, 80045-2581 CO USA; 2 Department of Cellular and Molecular Medicine, University of California San Diego, La Jolla, 92093-0021 San Diego, CA USA; 3 Department of Neurosciences, University of California San Diego, La Jolla, 92093-0021 San Diego, CA USA; 4 Rady Children’s Institute for Genomic Medicine, San Diego, 92123 CA USA

**Keywords:** Genomics, Brain development, Brain homeostasis, Genomic mosaicism

## Abstract

Genomic mosaicism describes the phenomenon where some but not all cells within a tissue harbor unique genetic mutations. Traditionally, research focused on the impact of genomic mosaicism on clinical phenotype—motivated by its involvement in cancers and overgrowth syndromes. More recently, we increasingly shifted towards the plethora of neutral mosaic variants that can act as recorders of cellular lineage and environmental exposures. Here, we summarize the current state of the field of genomic mosaicism research with a special emphasis on our current understanding of this phenomenon in brain development and homeostasis. Although the field of genomic mosaicism has a rich history, technological advances in the last decade have changed our approaches and greatly improved our knowledge. We will provide current definitions and an overview of contemporary detection approaches for genomic mosaicism. Finally, we will discuss the impact and utility of genomic mosaicism.

## Introduction

In the last ten to fifteen years, research efforts increasingly focused on genomic mosaicism—the phenomenon where individual or entire lineages of cells within a tissue harbor genetic mutations that are not present in every cell from one individual [1]. These genetic mutations are generally a consequence of non-fidelity during DNA replication or repair and can be caused by intrinsic or extrinsic mutational mechanisms [2–4]. If such an event occurs early in embryogenesis, the resulting variant will be present in multiple organ and tissue types throughout the body; however, if the variant occurs later after cell fate has already been determined, it will only be detected in that specific lineage [5, 6]. In the case of the nervous system, a mutation that arises from neural stem cells lining the neural tube early in development [7–9] would be present throughout all or many cells within the brain [5]. Alternatively, if a mutation occurs in a terminally differentiated neuron, that mutation will only be present in this one cell and persist for the remaining lifetime of the neuron [10].

Historically, studies devoted to mosaicism predominantly focused on its impact on human disease [1, 11]. This view is motivated by overgrowth syndromes and cancers that harbor mosaic ‘driver’ mutations that result in increased proliferation and are, consequently, positively selected. In some cases, they simply would be incompatible with life if present in every cell of our body: examples are the overgrowth disorder Proteus syndrome or focal brain malformations [12–17]. On the flip side, disorders where classical *de novo* mutations are typically causative—such as autism spectrum disorders (ASD) or constitutive malformations of cortical development (MCD)—can also be caused by mosaic mutations [18, 19]. While reported examples are often related to systemic and quite abundant mosaicism, the potential role of this phenomenon if ‘hidden’ within a tissue at lower levels is intriguing; this has been discussed and explored specifically for neurological and neuropsychiatric disorders by the NIH-sponsored Brain Somatic Mosaicism Network (BSMN) [20].

More recently, however, we increasingly appreciate mosaicism as a natural phenomenon that occurs throughout development and in every aging cell [21, 22]. This view highlights the utility of genomic mosaicism in understanding lineage development and aging homeostasis of cells—through lineage tracing and mutational signature analysis [23–26]. Although the vast majority of mosaic variants may not be drivers of disease, they can nevertheless help us to understand pathologies as readouts of mutational exposures or clonal distributions.

Here, we will provide an overview of these topics, definitions that we currently employ to categorize and understand mosaicism, and a summary of the most commonly used approaches to detect genomic mosaicism. Finally, we will also contrast the use of ‘natural’ with ‘engineered’ mosaicism and highlight their advantages and disadvantages. While we will provide a discussion of more general aspects of genomic mosaicism, most examples will focus on the mammalian brain. For a further discussion of this phenomenon in other tissue contexts or from a more technical perspective, please refer to reviews authored by us and others [27–33].

Also of note, our categorizations focus entirely on the nuclear genome and ignore genetic variation that is present in the mitochondria [34]. Because of the many copies of the mitochondrial genome in every cell and the phenomenon of heteroplasmy, each cell is mosaic for mutations in this organelle. While this is an important field of study, for this review, we will largely ignore this phenomenon. We point interested readers to excellent reviews and manuscripts of interest [34–38].

### Categorization of Mosaic Variants

While genomic mosaicism itself is defined straightforwardly by the presence of a genetic variant in some but not all cells within a collection, it can be further categorized in different ways. For instance, in clinical genetics, the patterns and impact of mosaic mutations are important [39], whereas in reproductive genetics the risk of transmission to the next generation is central [27, 40]. In the latter context, a mosaic variant can be inherited by the next generation if it arose before the specification of primordial germ cells—the progenitors of all germ cells—or within their lineage; recurrence risk within a family is consequently a result of the level of mosaicism that is present in the parental gonads [6, 27, 41].

**Fig. 1:**
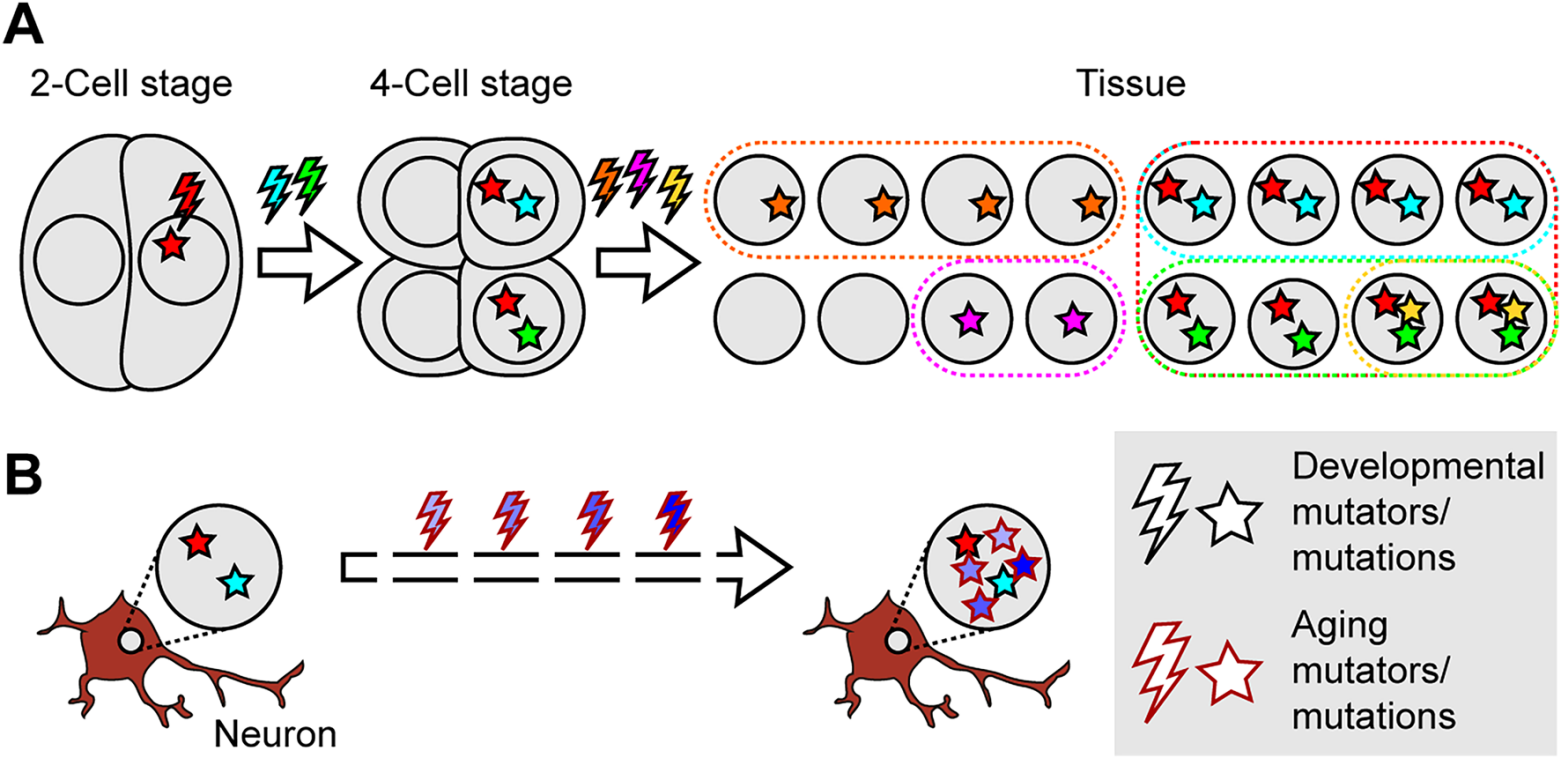
Developmental and aging mosaicism. **A** In development, mutations that occur at very early stages are transmitted to daughter lineages. Subsequent mutations further distinguish distinct lineages or sub-lineages. Developmental mutations are obligatory clonal mosaic variants. **B** Differentiated cells (here exemplified by a postmitotic neuron) already carry developmental mutations and accumulate additional aging mutations. In postmitotic cells, these aging mutations are obligatory private mosaic variants. Note that developmental and aging mutators are primarily distinguished by their timing, but may share intrinsic or extrinsic mutagenic stressors.

For developmental analyses, the classification of genomic mosaicism can derive from the timing of mutations within a lineage. For instance, if a somatic mutation arises in a proliferating cell, all daughters of this cell will inherit the variant [42]. This is often referred to as ‘clonal’ mosaicism, and it is contrasted with ‘private’ mosaicism which is only present within one cell and is often a consequence of cellular aging [43]. An alternative but related classification distinguishes between ‘developmental’ and ‘aging’ mutations (Fig. 1). The former are obligatory clonal mosaic variants, whereas the latter are private if they occur in a terminally differentiated, postmitotic cell, such as a neuron. However, an aging mutation in a proliferating cell would fulfill the definition of being clonal. While the theoretical distinction of these classes is relatively straightforward, experimentally, it is limited by the detection sensitivity of the employed analytical method. Therefore, the concept of ‘detectable’ clonal mosaicism is sometimes employed for the analysis of entire or larger subsets of tissues, typically synonymous with early developmental or extensively selected mosaic variants [42, 44].

An important feature of mosaic variants when analyzed in the context of tissues or a collection of cells is the Allelic Fraction (AF; also used with a preceding variant, VAF, alternate, AAF, or minor, MAF). This metric describes the fraction of mutant alleles relative to all detected alleles. For instance, a germline heterozygous mutation is detected at an AF of 0.5 in every tissue. In contrast, mosaic variants are detected at lower AFs that reflect their abundance: a mutation that occurs in one of two diploid cells would be present at an AF of 0.25, whereas one that occurs in one of 50 diploid cells at an AF of 0.01. To obtain the fraction of cells harboring a mosaic mutation, for diploid cells, one has to multiply the AF by two; for haploid cells, the AF is equal to the cellular fraction.

### Types of Mosaic Variants

**Fig. 2:**
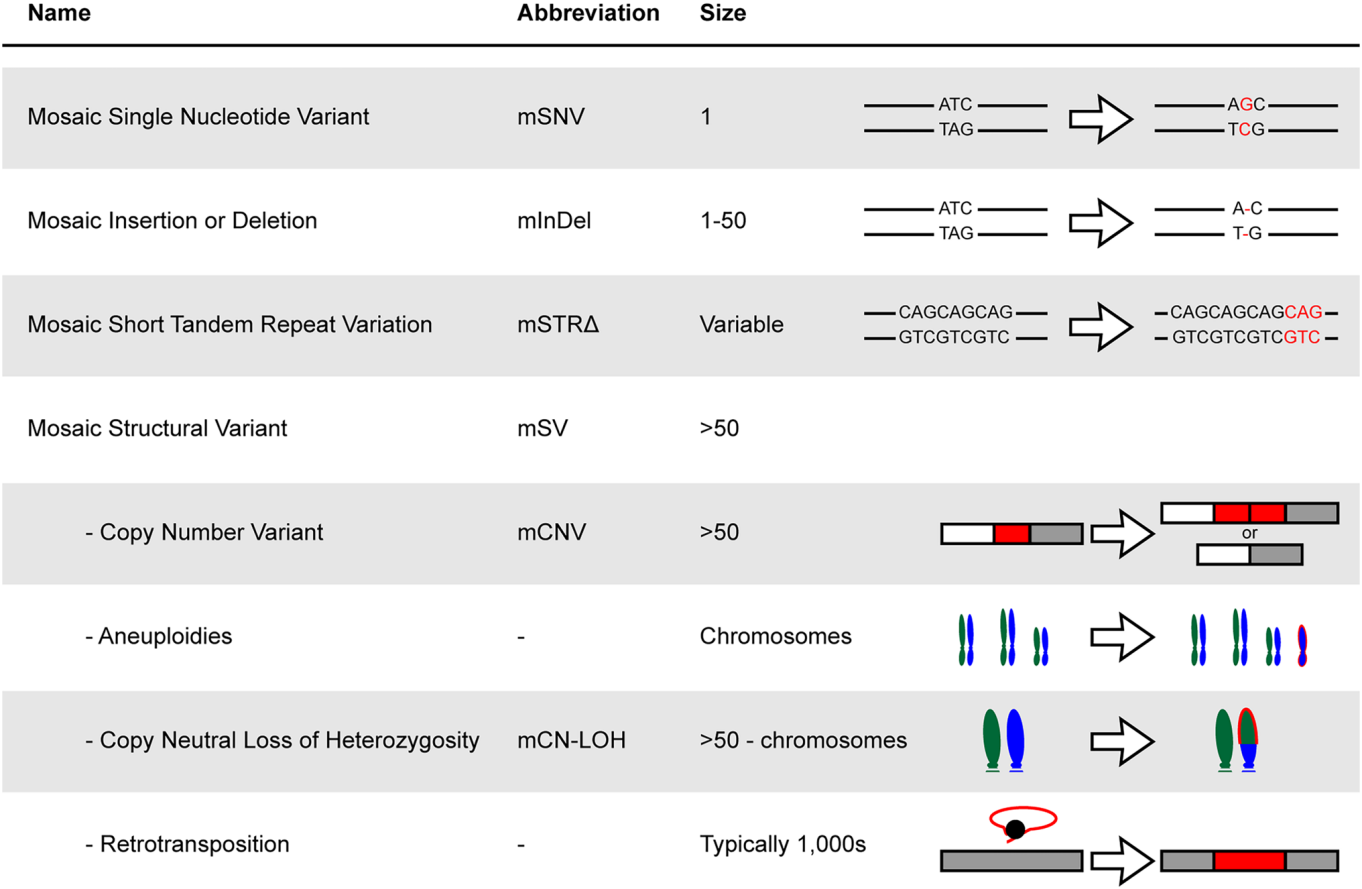
Types and Scale of Mosaic Variants. Small mosaic variant types like mSNVs, mIndels, or mSTR∆s are the most common types of mosaic genetic variation. However, larger mosaic variants can be grouped as mSVs. Each type of observed mosaic variant is illustrated in this figure with an example. mSNV: a T to G base pair substitution; mInDel: a one-base pair deletion; mSTRΔ: a one unit CAG expansion; mCNV: two examples for a genomic tandem duplication and a deletion; aneuploidies: duplication on one chromosome; mCN-LOH: duplication of a part of the green haplotype while partially losing the blue haplotype; retrotransposition: insertion of the red retroviral mRNA sequence into the locus.

Independent of their timing, mosaic variants are also distinguished based on the type of mutations (Fig. 2); and these are largely similar to variants that are encountered as germline mutations [45–49]. The most commonly detected and conceptually simplest type of mosaic variants is mosaic single nucleotide variants (mSNVs). They encompass any variant that exchanges one base for another and are typically a result of DNA damage that is not or inadequately repaired [22, 50]. While all possible transitions and transversions can be observed in the genome, intrinsic and extrinsic mutational mechanisms result in significant biases in their rates [51]. For instance, the transition from a cytosine to a thymidine (denoted as C>T) is often encountered at high frequencies, as it results from the deamination of a methylated cytosine [52]; this, in turn, is read as uracil during replication, fixing this change in the genome. As cytosine methylation and the subsequent deamination are a relatively common occurrence in the human genome, this is an often encountered mosaic mutation type. While these concepts have been described in much more detail for various cancers [51, 53–55], they are now an integral part of any mosaicism research.

The burden estimates of mSNVs vary depending on the experimental design and employed method. A study by Bae and colleagues proposed that neuronal progenitors accumulate 5.1 mSNVs per neuronal progenitor per day which culminated in a newborn neuron harboring 300-900 mSNVs within one year of birth [21]. This was in agreement with studies by Lodato and colleagues who also noted that postmitotic neurons accumulated dozens of more mutations per year with some variability depending on the brain region [26]. While these estimates are subject to potential technical artifacts that may increase false-positives and suffer from false-negative mSNVs, they are comparable to mosaic mutation accumulation in clones isolated from somatic proliferating tissues [56]. An interesting comparison benchmark for these rates is the human per-generation mutation rate, which mostly reflects mutations accumulated in the lineage of the egg and sperm [27, 57, 58]. Here, the male contribution increases by approximately 1.5 variants per year, which is comparable to the rates found for clones within the seminiferous tubules [56, 59].

The functional consequence of mSNVs is dependent on their location, and their interpretation can be challenging if they do not directly impact the function, folding, or expression of a protein product [60, 61]. In addition to the ambiguity of the mSNV’s effect, it also has to be put into the context of its abundance and presence in various tissues, which can further modify the functional impact (a consideration that also applies to other types of mosaic mutations). While the most abundant type genome-wide, most mSNVs will have no or limited impact on phenotypes.

A variant type that is conceptually closely related to mSNVs is small mosaic insertions or deletions (mInDels). They range by definition from 1 to 50 base pairs in size and are often the result of polymerase slippage or imperfect repair [62]. In normal development, they likely occur an order of magnitude less frequently than mSNVs based on population frequency and mosaicism assessments by us and others [23, 44, 59, 63]; of note, these analyses may underestimate their frequency due to increased technical challenges in detecting mInDels compared to mSNVs. Their functional impact is higher than for mSNVs when found in coding sequences, as they often result in a frameshift mutation that results in the premature termination of a protein.

From a mosaicism perspective, short tandem repeat expansions and contractions (mSTRΔs) represent a largely unexplored territory [46]. They are generally a result of polymerase slippage in mitosis, although unequal crossing over in meiosis also plays a role in their origin; yet, the latter is irrelevant for somatic mSTRΔs, as they occur in non-meiotic tissues, such as the brain. While we previously reported genome-wide mSTRΔs in the sperm of fathers that were identified leveraging variation data from offspring [6], a more comprehensive analysis of these mosaic mutations across tissues has not been performed—mainly due to technical challenges when using typical sequencing techniques [64]. Thus, their frequency is largely unknown, despite their potential health impact being well-understood for a range of neurological disorders [65, 66]. For instance, the classical repeat disorder Huntington's disease has been reported by Telenius and colleagues to exhibit tissue-specific instability—or mosaicism—in the brain decades ago [67]. Based on their work, a mosaic increase in expansions may worsen local tissue phenotypes.

A less frequent form of mosaic variants is mosaic structural variants (mSVs). They can be copy-neutral or result in copy number-variation (mCNV); mCNVs, like germline CNVs, are defined as genomic intervals with deletions or duplications—as small as a few hundred base pairs or as large as entire chromosomes, which are referred to as aneuploidies [68]. Depending on the type of mSV and the employed detection method they can be challenging to detect; yet, they were successfully identified in individual cells or the context of disease if abundantly present [69–72]. Furthermore, a recent study suggests that as many as 10% of neurons may carry mCNVs and in some cases complex karyotypes [73]. While they are significantly rarer events than mSNVs or mInDels, they may have a much larger potential impact on function, largely depending on their size, affected genomic regions, and whether they result in a change of copy number.

One interesting sub-class of mSVs that has received increased recent scrutiny is mosaic copy number neutral losses of heterozygosity (mCN-LOHs) [74–76]. Loss of heterozygosity refers to a phenomenon where, instead of carrying paternal and maternal genomic material, two copies of one or the other are present. These are referred to as ‘copy number neutral’, as there are still two alleles, and the region is still considered diploid, despite receiving two copies from one parent [49]. These mSVs can range in size from very small genomic intervals to entire chromosomes. mCN-LOH variants have been studied in the field of cancer genetics and the context of clonal selection—as a LOH event could alter a heterozygous cancer-driving variant to a homozygous state [49, 77, 78]. However, in cases of severe mutations, mCN-LOHs may also be selected to express milder phenotypes, as demonstrated by an intriguing study by Lee and colleagues [79].

There is one additional variant type—technically a sub-class of mSVs—that leads to genomic mosaicism and is significantly enriched in brain tissue: retrotransposition of mobile elements. The most commonly studied type is LINE-1 or L1: L1-related sequences comprise 17% of the human genome and the L1 family contains the most active transposable element in the human genome [80, 81]. These retrotransposons can create mosaic populations by inserting DNA sequences in random locations in the genomes of different populations of cells. While largely quiescent in most somatic cells, it has been shown that LINE-1 elements are highly active in developing neuronal progenitors [82, 83]. There has been disagreement regarding the frequency of these events, and current estimates range from <0.6 to 13.7 insertions per neuron [84, 85]. As a potentially relatively large insertion (thousands of base pairs), these events may impact gene expression significantly; this is compounded by their potential to interfere with splicing if integrated into an intronic region [85]. In addition, they are also interesting functionally from an evolutionary viewpoint as they quickly enable sequences to be transcribed and expressed [86]; however, whether an analogous mechanism is important in the context of brain mosaicism remains currently unexplored.

### Detection of Mosaic Variants

**Fig. 3:**
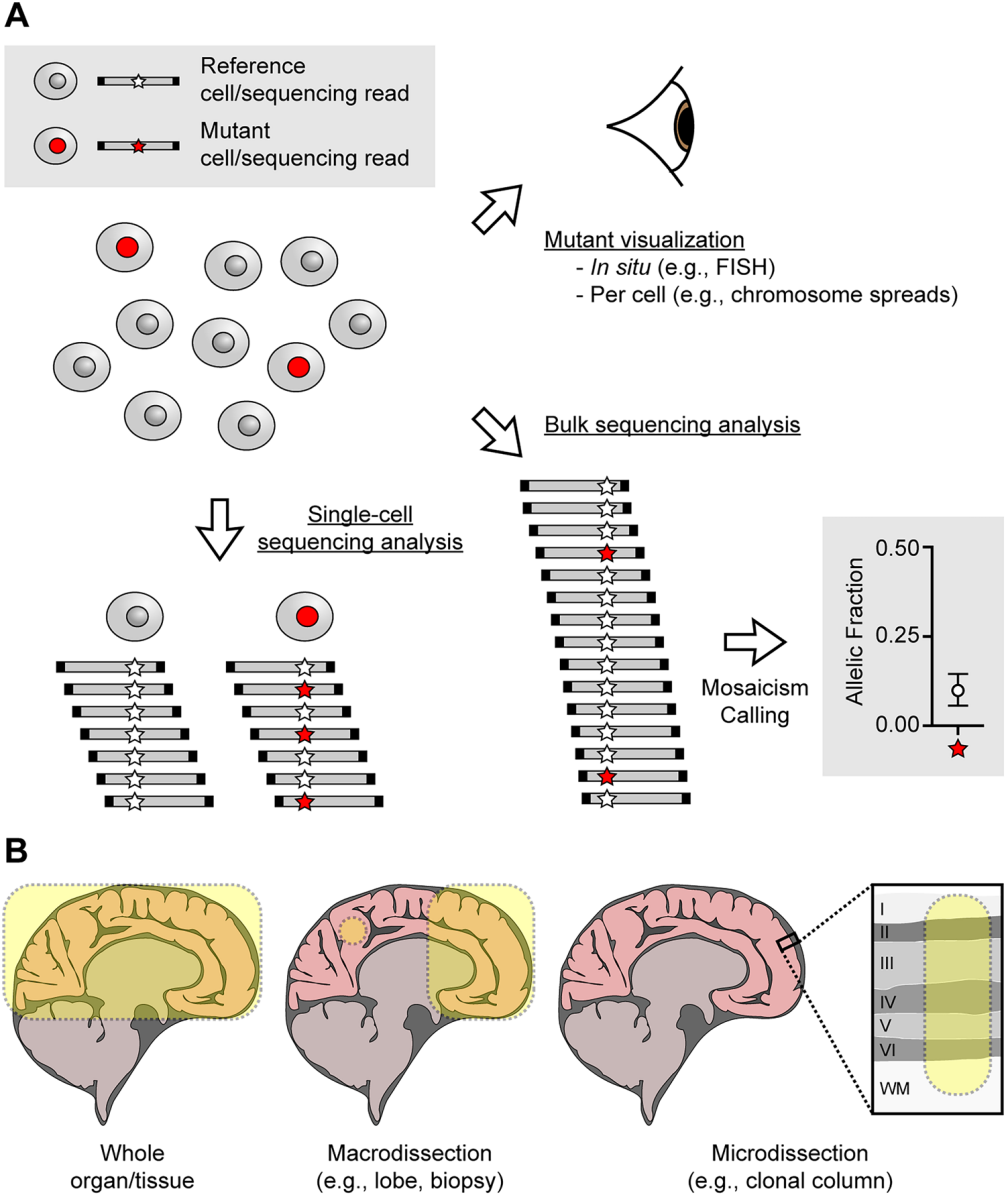
Types and scale of mosaicism detection approaches. **A** Mosaic mutations in a subpopulation of cells may be detected by three theoretical approaches: (1) through direct visualization of mutations employing FISH or chromosome spreads, (2) through bulk analysis of genomic material, or (3) through assessment of genomic material at the level of single cells. **B** Biological insights obtained from mosaicism analysis are heavily dependent on the scale of sampling. For instance, mosaicism may be detected from an entire tissue like the neocortex, microdissection, or microdissections, all of which provide distinct information due to their drastically different scale.

For this review, we distinguish three distinct types of mosaicism detection (Fig. 3A, Table 1): (1) visualization of mutations in tissues or on the level of individual cells; (2) bulk genomic DNA analysis where mutant alleles are detected using specialized mosaicism detection tools; and (3) single-cell genomic DNA analysis. For the latter two approaches, the most common choice of technology is direct sequencing analysis. Mosaicism analysis can be performed for the detection of unknown or already known variants; this is true across the three mentioned types.

**Table 1:** Approaches to discover novel mosaic variants

Approach			Resolution	Advantages	Limitations
Top-down – bulk tissue analysis			–	Comprehensive for abundant variants	Sensitivity is limited by read-depth; and highly dependent on the sampling strategy
*Methods (Detection)*					
DNA microarrays		mSV		Low cost; high depth and sensitivity	Limited resolution based on array density
Whole-exome or panel sequencing		mSNV		Lower cost than genome sequencing; higher read-depth and sensitivity in practice	No analysis outside the exome (e.g., introns) or panel; capture/enrichment can cause artifacts
Whole-genome sequencing		mSNV-mSV		Comprehensive variant detection	Highest cost results in lower read-depth and sensitivity in practice
RNA sequencing		mSNV		Lower cost than genomes; captures functional information	No analysis outside of expressed genes; RNA editing/processing may introduce artifacts or false positives
Top down – clonal biopsy analysis –				Highly sensitive for clonal variants; the sweet spot for many applications	Less comprehensive than bulk tissue analysis and less sensitive than single-cell analysis; relies on a clonal architecture of tissues for maximum efficiency
Bottom Up – Single-Cell Analysis –				Sensitivity down to a single-cell level	Lower sampling due to cost (one analysis per cell) can prevent comprehensive sampling; amplification artifacts
*Methods (Amplification)*					
Enzymatic whole-genome amplification		–		Possible on extracted cells or nuclei from primary tissue that was frozen	Introduces amplification artifacts and consequently false positives
Clonal expansion		–		Relatively cheaper; higher fidelity to the endogenous replication machinery	Cell culture artifacts; require living cells that can clonally expand

In the realm of visualization of genomic mutations, chromosomal karyotyping—in use since the 1950s—is a way of imaging entire chromosomes following an arrest in metaphase [87]. With the addition of techniques such as G-banding, it is possible to identify mutations at the partial-arm or whole chromosome level [88]. While often used in clinical genetics to infer karyotypes or large-scale structural variants for a patient, these methods inherently work on a single-cell level. Thus, this renders them excellent tools for understanding genomic mosaicism if a sufficient number of cells are assayed. Indeed, this approach allows for the identification of constitutive as well as mosaic deletions, duplications, or translocations of sufficient size [88, 89].

Employing RNA or DNA fluorescent probes, Fluorescent *in Situ* Hybridization (FISH) allows the sequence-specific detection of chromosomal locations in interphase and metaphase [90–92]. Thus, using FISH, researchers and clinicians can assay specific intervals on chromosomes for aneuploidies [93, 94]. FISH can also be used in the context of a tissue [95]; combining single-molecule FISH with allele-specific probes theoretically also allows the assessment of mSNVs if they are located in expressed genes [96, 97]. While not currently employed, further technological advances and the combination with super-resolution microscopy may eventually enable the direct detection of genomic mosaic variants.

While the previous methods are useful for interrogating mSVs visually at low throughput, they are generally not very scalable. In the 2000s and 2010s, DNA microarrays were popularized as a higher-resolution method to interrogate DNA copy numbers [98, 99]. Array comparative genome hybridization (aCGH) panels were designed so that an experimental sample would be compared against a diploid reference sample, and these arrays were further improved by the addition of single nucleotide polymorphism (SNP) genotyping panels that could also identify LOH. This approach vastly improved the ability to detect mCNVs and mCN-LOH down to a resolution of approximately 50 kb at AFs as low as 0.01 from bulk samples [100–102]. Such SNP genotyping data was leveraged effectively to detect these types of mosaic variants in blood, which allowed the detection of clonal hematopoiesis and its impact on neurological disorders [71, 78, 102, 103]. While this approach still has limited resolution as compared to next-generation sequencing technologies, DNA microarrays remain relevant due to the still competitive cost for large-scale genomic analyses.

### Detection of Genomic Mosaicism from Tissues

The advent of next-generation sequencing (NGS) fundamentally changed our approach to detecting mosaicism [104]. As NGS inherently is a method that assesses the sequence of individual DNA molecules, it is uniquely suited for mosaicism research—and it allows the detection of mSNVs. The theoretical limit of sensitivity for mosaic variant detection is set by the depth of sequencing and statistical considerations of random sampling. For instance, employing simple binomial calculations, a variant present at 0.1 AF in a non-limiting DNA sample will be picked up in at least one read 65.13% of the time when sequencing at 10×, or 99.99% at 100×. Similarly, a variant present at 0.01 AF will be picked up only 9.56% of the time at 10×, or 63.40% at 100×.

As the employed read-depth is typically cost-limited by the sequenced genomic space, whole-genome sequencing (WGS) is generally performed at lower depths (~30–60×) than whole-exome sequencing (WES; ~50–100×) or targeted panels (often >1,000×) [105, 106]. Due to decreasing sequencing costs, these numbers are very much moving targets, and we and others have employed deeper sequencing to understand mosaicism at higher sensitivities [23, 107–110]. This approach is especially powerful when assessing unbiased bulk mosaicism on a tissue level or within a microdissection (Fig. 3B). When dealing with microdissections, instead, it is common to perform regular or even shallower sequencing on a larger number of samples [24, 56, 111]. An alternative approach is mosaicism detection from RNA sequencing data [112]. Conceptually similar to detecting mosaicism from exome sequencing, there are biological and technical complications that need to be considered carefully, such as the level of transcription from areas containing mosaic variants, potential splice variants that may lead to uncalled mosaicism, or RNA editing that may result in false-positives. However, RNA sequencing analysis may reveal additional functional details of the impact of mosaic variants as DNA is transcribed to mRNA or even downstream (Table 1).

While there is a strong correlation between read depth and sensitivity, the above-provided sensitivity calculations assumed that one mutant read is sufficient to detect mosaicism. In practice, NGS and its computational processing have inherent error rates that impair our ability to detect mosaic variants [29]. Thus, in addition to sequencing at sufficient depth, mosaicism analysis also requires specialized analytical pipelines [113]. As unbiased mosaicism analysis from bulk sequencing samples was pioneered by the cancer research field, variants were typically expected to be positively selected, consequently at relatively high AFs, and only present in the tumor but not ‘normal’ control tissue. Thus, algorithms were initially designed to detect mosaicism for this purpose specifically [17].

More recently, the focus of mosaicism research has shifted to include lower abundance mosaic variants (<0.05 AF) and those that are shared among tissues. In many cases, these modern pipelines still include classical tools, such as Mutect2 [114, 115], but also employ additional classifiers that provide a secondary level of evaluation and increase specificity (i.e., reduce the number of false-positive mosaic variants) [116–118]. Alternatively, some programs provide both variant detection and classification, such as our previously developed tool MosaicHunter [119]. All of these pipelines typically have areas of strengths and weaknesses, and they might require different experimental designs (e.g., a tumor-normal comparison). Therefore, many analytical pipelines employ a combination of methods to improve sensitivity, specificity, or both. If a variant is known (or a group of variants is routinely seen), these approaches are often replaced by more specialized pipelines (e.g., for drivers of clonal hematopoiesis) [120]. It is important to note that these analytical approaches are rapidly evolving in parallel with sequencing technologies and computational innovations. For instance, duplex sequencing can significantly improve the specificity of any detected variants but comes at the cost of increased sequencing depth requirements [121, 122].

An important part of many mosaicism detection experiments from bulk samples is the subsequent validation of candidate variants through orthogonal approaches. We want to focus on two of the most common here: targeted amplicon sequencing and droplet-digital polymerase chain reaction (ddPCR); we do, however, acknowledge that there are many others, such as subcloning of amplified products, Multiplex-Ligation Probe Amplification (MLPA), denaturing high-performance liquid chromatography (DHPLC), and so forth [19, 123, 124]. The use of targeted amplicons that are subsequently sequenced at high depth is a commonly used approach for validation and quantification by us and others [6, 125, 126]. ddPCR enables the genotyping of single molecules through lipid droplet partition [127]. While the latter has inherent advantages, such as being independent of NGS approaches and highly sensitive, it is also relatively expensive when not used to test the same variants repeatedly and less scalable. Similar to the unbiased detection methods, the use of validation approaches is dependent on the specific question, the number of variants tested, and similar considerations.

### Detection of Genomic Mosaicism from Individual Cells

Finally, the detection of mosaicism can also be performed on a single-cell or single-nucleus level (Table 1). Technological advances now allow the high-fidelity amplification of a single genome and the detection of mSNVs [128, 129]. This has been used to great effect for neurons to understand development, aging, and disease or in cardiomyocytes [25, 26, 130]. Similarly, employing whole-genome amplification, mSVs can be detected from a single cell; this has been extensively studied in sperm but also in neurons [73, 131–134]. A variation of this approach is the analysis of a clone (e.g., crypts in the gut) *in situ*, where it is possible to isolate tissues that are mono- or low-level polyclonal. This allows an understanding of mosaicism across phylogenies but also within human tissues to understand mutation rates in such clones [24, 56, 135]. A different and interesting addition to single-cell or single-nucleus technologies is the combination with functional readouts, such as single-cell RNA sequencing [136].

If a cell type is capable of clonal expansion (e.g., skin cells, neural progenitors, cancer cells), it is possible to expand individual cells and sequence the resulting population as a representation of the genomic mosaicism present in the founder cell [21, 137, 138]. This takes advantage of the superior amplification of genomic DNA by the cellular machinery. For cell types that are not inherently available for clonal expansion, it is possible to perform nuclear transfer into proliferation-competent donors; this has been demonstrated for postmitotic neurons in mice [139]. However, this is a complicated process that has not yet been successfully applied to human neurons.

While it is inherently attractive to assess mosaicism on the level of a single cell, there are some technical limitations to employing this approach. First, the amplification of genomic material from a single cell or nucleus is error-prone and may result in a larger number of false-positive mosaic variants [140]. There are, however, strategies to remedy this, such as the genotyping of additional material for confirmation of clonal mosaicism or the restriction to ‘phased’ haplotypes [110, 141]. Here, the assumption is that mosaic variants should be restricted to the one parental haplotype where it originally arose. If a mosaic mutation is instead found across the two, it should be considered an artifact, as it is exceedingly unlikely for the same mosaic mutation to occur twice within a sample or cell [29, 116]. Similarly, clonal expansion—if possible—may suffer from cell culture artifacts, such as mutations acquired after isolation from primary tissues or selection of certain genotypes. Second, independent of possible errors, there is an additional conceptual limitation for employing single-cell analysis. We often refer to this approach as ‘bottom-up’—in contrast to the bulk-based ‘top-down’. While single cells offer the highest sensitivity of mosaic variant detection, they also provide a less comprehensive picture of clonal mosaicism depending on the sampling strategy and the number of assessed cells.

### Impact and Utility of Genomic Mosaicism

**Fig. 4:**
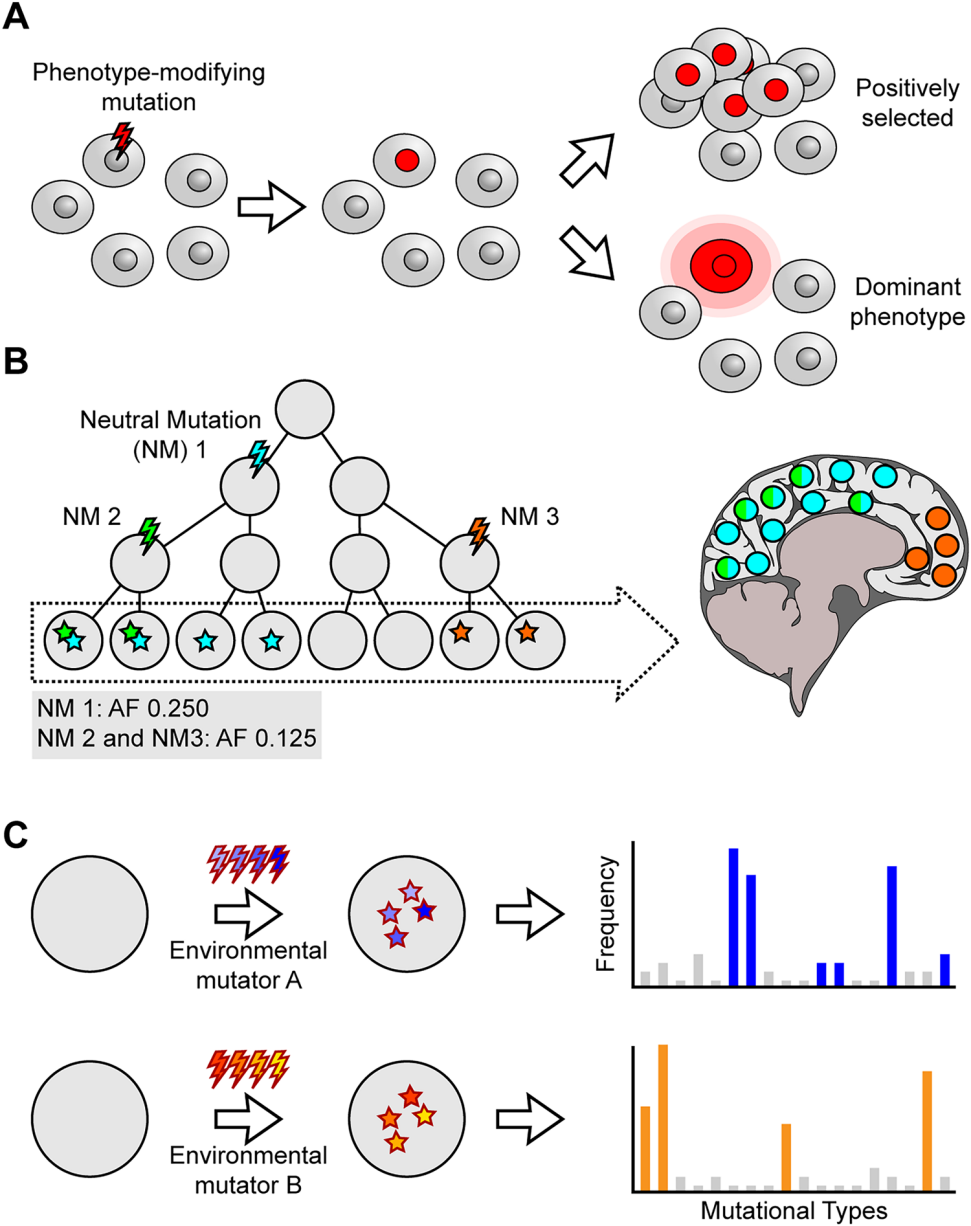
Impact and utility of natural mosaicism. **A** Mosaic mutations may act as a driver of disease. Clones harboring mosaic mutations can be positively selected for continued expansion and proliferation which may directly result in disease. Alternatively, mosaic mutations may exhibit a dominant phenotype. Note that these two scenarios are not mutually exclusive.** B** Natural mosaicism marks cellular lineages and can be used for lineage reconstruction or clonal analysis. For instance, in this example, distinct clones are marked by Neutral Mutation (NM) 1 and NM 3, whereas NM 3 marks a sub-clonal lineage in combination with NM 1.** C** Natural mosaicism can be used as a molecular readout of the microenvironment that cells are encountering. Exposure to different environmental mutagens such as reactive oxygen species or toxins can lead to very specific ‘mutational signatures’.

As discussed in the preceding sections, various types of mosaic variation can be detected through different technical and conceptual approaches. However, why do we want to detect mosaicism in the first place? We propose that mosaicism detection can serve three fundamental purposes. (1) Genomic mosaicism may have a direct impact on observed phenotypes (Fig. 4A); this can be either due to positive selection of the mutation and a change in clonality or due to a dominant phenotype. Thus, the detection of these mutations may aid our understanding of disease pathology or enable treatment in the future. In addition, somatic mosaic variants can also be used for their utility: (2) as clonal lineage marks to understand normal development or mutational rates (Fig. 4B); (3) as a readout of environmental processes that induce certain types or patterns of mutations at developmental or past cellular time points (Fig. 4C). We will discuss each of these three with a focus on the brain subsequently; however, we will not provide a comprehensive discussion of each point for brain mosaicism. Thus, we want to draw attention to other excellent reviews that highlight these concepts in the brain, especially in the context of diseases [10, 18, 72, 142–146].

Mosaic variants within the nervous system have been identified as drivers of neurological disease in several instances. For instance, focal cortical dysplasia (FCD) and hemimegalencephaly (HME) are classical mosaicism-driven neurological diseases; they are characterized by dysmorphism and hyperexcitability of a small region of the cortex or an entire cerebral hemisphere, respectively [147]. Analysis of brain tissues from FCD and HME patients has revealed activating somatic mutations in the *mTOR-AKT3-PI3KCA* pathway as well as loss-of-function mutations in genes that are negative regulators [13, 16, 17, 148–151]. Importantly, these somatic mutations were mostly found exclusively in brain tissue, suggesting that they were acquired later in neurodevelopment [152]. Conceptually, these disorders are part of a spectrum where the exact phenotypic presentation depends on the timing of the driver mutation and its abundance within the tissue. Mechanistically, they likely represent a combination of positively selected and dominant mechanisms, as there is evidence of associated overgrowth syndromes and a patch of hyperexcitable dysplastic cells may induce drug-resistant epilepsy within a network of neurons.

In other neurological disorders, such as Alzheimer’s disease, schizophrenia, or ASD, it is speculated that mosaic variants may also contribute to or exacerbate the overall phenotype [71, 107, 108, 153–157]. While the contribution of mosaicism to such disorders is clear in cases where it is also detectable from blood (especially if a known disease mutation), the direct contribution of ‘cryptic’ (i.e., brain-specific) mosaicism has been more elusive. Unlike the clear disease mutations found at lower abundance (often below 0.10 or even 0.05 AF) in FCD [151], the minimum abundance of causative mutations in other disorders remains unclear. Thus, this is still an exciting ongoing field of research.

### Utility of Natural Genomic Mosaicism for Lineage Tracing

Just as the timing of mosaic mutations in development is important to understand disease pathogenesis, researchers can also utilize neutral somatic mutations to study developmental processes and clonal lineages directly (Fig. 4B). Indeed, analysis of naturally occurring somatic mutations as lineage ‘barcodes’ has been utilized to study embryonic development and more generally cellular lineages of humans [24, 158]. We and others have similarly employed this framework to specifically understand these in the brain [23, 25, 110, 138]. An important addition to such studies—independent of the use of bulk or single-cell analyses—is the restriction of analysis to specific cell types (often neurons). This is typically achieved through fluorescence-activated nuclear sorting, as most human brain samples are frozen immediately after collection, which significantly complicates cellular sorting [159].

What are some of the insights that have been derived from employing mosaicism for lineage analysis? For instance, work by us and others has focused on the spread of clones that can be distinguished by mosaic variants [23, 110]. In 2015, the Walsh Lab sequenced 36 single-cell neurons to identify thousands of somatic mutations [25]. From these, they were able to reconstruct a lineage tree and identify points of divergence. As an extension, Bizzotto and colleagues performed high-depth sequencing on multiple human tissues to identify mSNVs [110]. They concluded that at the onset of gastrulation, there exists a pool of approximately 170 cells with 50 to 100 founders committed to the forebrain. These analyses also revealed that the spread of clones across the cortical surface is largely inverse-correlated with the observed abundance, although there are exceptions to this. A recent study by us further revealed that within the neocortex—in contrast to the overall patterning of the neural tube—clones are first separated along the left-right axis before anterior-posterior [23]; however, the same left-right separation did not extend to the hindbrain. Focusing on the neocortical hemispheres, we further proposed a neural progenitor founder pool of approximately 90 to 200 cells at the time of left-right separation.

Clonal analysis that focused on lineages of defined cell types further confirmed previous findings from rodent models for the first time in humans [23, 110, 136, 160–163]. These studies also suggested the existence of developmental bottlenecks or restrictions that can modify the contributions of early lineages within similar tissues in the absence of selection. Together, while these studies described some aspects of neurodevelopment comprehensively, there are still many remaining questions that will require careful experimental (or sampling) design and analyses; these include the migratory and developmental patterns of interneurons, the lineages and clonality of microglia, and the developmental trajectories of non-neocortical brain regions.

### Utility of Natural Genomic Mosaicism to Map Mutational Histories

Finally, genomic mosaicism can also be a useful biomarker of cellular environment or stressors (Fig. 4C). The rate or frequency of mosaic mutations may reflect endogenous or exogenous mutators. For instance, the somatic mutation rate is significantly increased in neurons of individuals with mutations in the DNA repair machinery during aging [26]. The mutation burden can also be increased in seemingly healthy individuals in both germ cells and neurons due to alterations in the same or similar pathways [108, 164]. This approach even enables a distinction of repair fidelity across development: for instance, the earliest cell divisions in an embryo appear to show increased mutation rates compared to the latter, possibly due to the inheritance of the repair machinery through the egg cell [6, 44, 165].

Beyond the frequency of mutations, the observed types vary based on the mutator as well. Here, mSNVs are mainly analyzed and categorized from the perspective of the pyrimidine base (i.e., cytosine or thymidine) and the newly acquired mutations: thus, there are six possible substitutions, three for each. We mentioned above C>T substitutions and that they mainly derive from the deamination of methylated cytosine [52]; similarly, other cellular processes may drive different types of mutations. These patterns mainly derive from cancer studies but have been widely applied across genomic mosaicism research [166].

In addition to the described six categories of mutational types, more recently, the context of a mutation—the neighboring bases—has also been considered. This represented a significant innovation, as the two bases immediately adjacent to the mutated base allow for a finer dissection of molecular mutation mechanisms [167–170]. This is achieved through the statistical isolation of ‘mutational signatures’ which represent distinct potential drivers of mutations. While some of these turned out to be artifactual, others have been directly connected to internal and environmental mutagens, such as DNA replication, ultraviolet A light radiation, tobacco smoke, or certain chemotherapeutics [171–173]. An important limitation to assessing mutational signatures is the requirement for a sufficient number of observed mutations. Thus, this approach is mainly applicable to large collections of cancer genome data, or studies that leverage the individual genomes of cells like those centered on neurons [51]. In certain situations, lower numbers may be sufficient if driven by specific mutations, as demonstrated by a study focused on transgenerational mutation rates [164]. If this method can be implemented, it opens a window into the experienced environment of cells during development or in the context of disease.

For instance, neuroinflammation is a common symptom of many neurodegenerative disorders and is associated with a dysregulation of redox balance in the brain [174, 175]. A higher level of reactive oxygen or nitrogen species can result in elevated rates of somatic mutations in individual cells or their lineages present in the brain [176–178]. Analyzing the rates and types of somatic mutations in neurotypical versus diseased individuals provides information on the disease environment and may even identify contributing factors. In a recent example, this approach was applied to Alzheimer’s disease, confirming an inflammatory environment that causes oxidative DNA damage in neuronal nuclei [179]. The human genome effectively acts as a tape recorder of its environment; when combined with developmental lineage analysis it is possible to also resolve the embryonic environment. However, an efficient implementation requires the generation of large data sets that allow for the stratification of distinct developmental stages at higher resolution. The National Institutes of Health have recognized this limitation and have started the Somatic Mosaicism across Human Tissues (SMaHT) network which was initiated in 2022 and plans to provide a database of human genomic mosaicism and related technological toolboxes.

### Utility of Engineered Genomic Mosaicism

Conceptually, both lineage analysis and mutational signatures represent a fascinating conundrum. For both, adult tissues—often from deceased individuals—are employed to understand embryonic processes that often occurred decades ago. While this allows insights into early development in the context of an organism—humans—that is otherwise intractable for such studies, it comes with some caveats. First, lineage reconstructions require many assumptions, including that mutations and lineages are neutrally selected and do not disappear; while this is mostly an appropriate approximation, it has been demonstrated to be inaccurate in some cases, especially in the context of the first cell divisions [110, 138, 180]. Second, due to the naturally determined rate of mutation and technical issues, lineage trees often remain incomplete. Thus, while these approaches allowed interesting and fundamental insights into neurodevelopment despite these limitations, there are clear advantages when mosaicism can be engineered rather than passively detected.

**Fig. 5:**
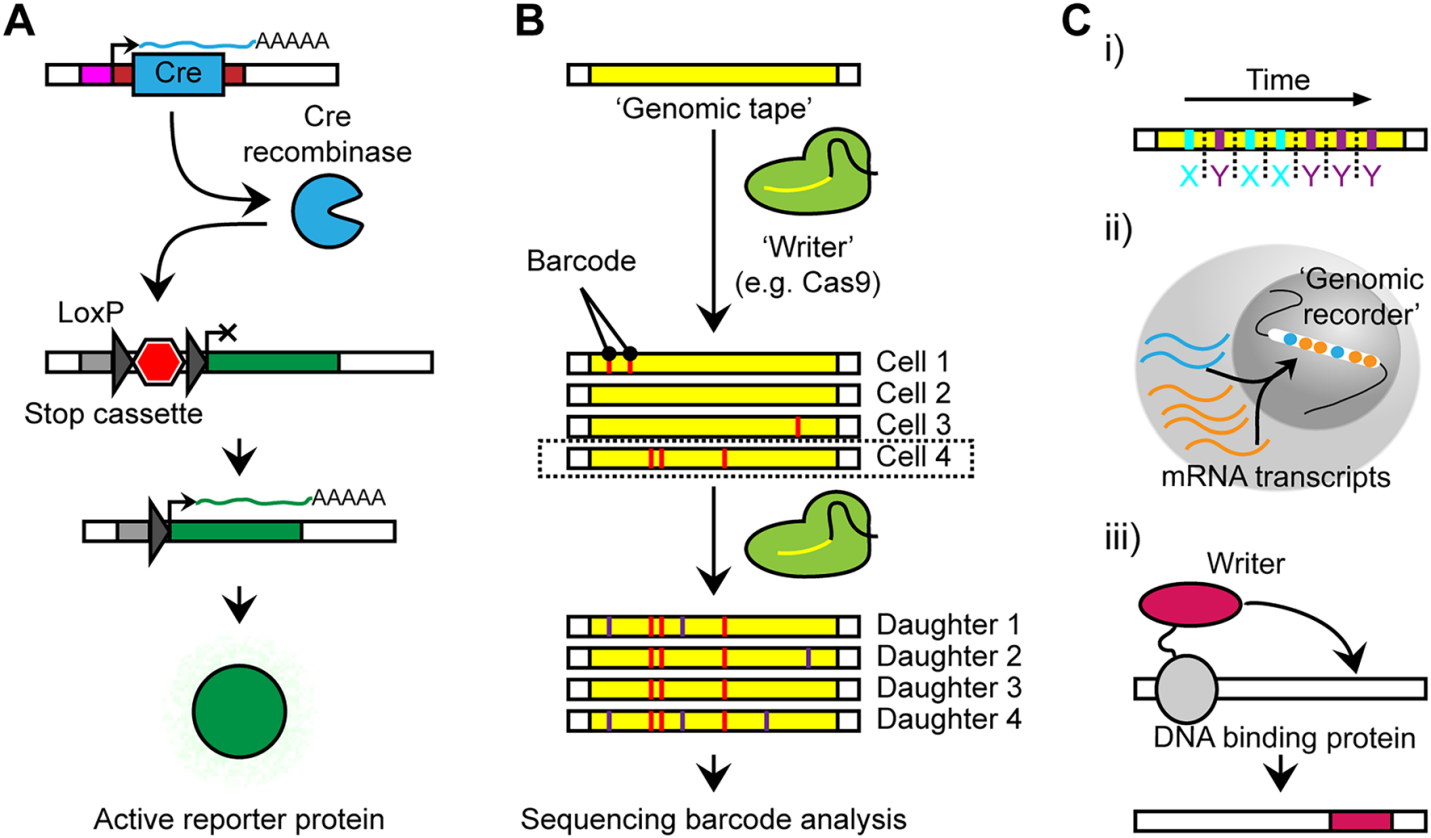
Types of engineered mosaicism. **A** One of the most utilized methods to track lineages involves the use of fluorescent markers, such as GFP. One possible configuration employs a stop cassette which is flanked by Loxp sites and prevents the transcription of GFP. When Cre recombinase is expressed from a lineage-defined locus, the stop cassette is removed and the GFP is expressed in this cell and its daughters. **B** A more recent innovation used to track lineages employs ‘genomic writers’ (e.g., Cas9) that are targeted to a defined locus, often denoted as ‘genomic tape’. Here these writers can introduce either random or defined mutations that act as genomic barcodes to distinguish cells and their lineages. These barcodes are subsequently read through targeted sequencing. **C** Using writers in combination with other systems, it is possible to further encode the temporal resolution of defined signals (i), the expression status of a cell (ii), or protein binding to genomic regions (iii).

Lineage tracing through engineered mosaicism has a long history in neuroscience research. Conceptually, the goal is to mark the genome of a subpopulation with a permanent change that can be detected at a later time point [181, 182]. For instance, classical lineage tracing experiments in the brain by Walsh and Cepko employed retroviral vectors where a subset of cells was labeled based on their location at the time of injection [183–185]. Depending on the vector, the readout of the mosaic change was based on a visual phenotype or a direct readout of a known genetic sequence. The advent of advanced mouse genetics enabled the now classical lineage tracing with a fluorophore or otherwise active reporter protein upon irreversible activation by a Cre recombinase (Fig. 5A) [181, 182]. More complex reporters include the use of combinatorial multi-fluorophore systems to differentiate individual lineages in parallel or Mosaic Analysis with Double Markers that sparsely label individual daughters of a single cell [186, 187]. The most significant drawback of this system for lineage tracing is the limited resolution employing fluorophores.

A potential solution is the direct readout of genomic alterations that can distinguish many lineages in parallel based on combinatorial sequence variation. The fundamental idea of such systems is the use of a genomic ‘writer’ with a controlled or known expression that can introduce genomic changes in a locus that contains ‘genomic tape’ (Fig. 5B). In 2016, a novel method named GESTALT (genome editing of synthetic target arrays for lineage tracing) utilized CRISPR/Cas9 to barcode cells in this way throughout early development [188]. This technique allows for the sequencing at a later developmental point and the reconstruction of a lineage tree by analyzing the barcoded sequence. A fundamental limitation of GESTALT is the introduction of random insertions and deletions that may interfere with each other and complicate lineage reconstruction. This was addressed by work from the same laboratory through the use of sequential genome editing in a proof-of-concept in cell lines [189]. A similar approach has also been employed in mice [190].

Employing an orthogonal but related concept, Kalhor and colleagues devised a homing CRISPR method in which homing guide RNAs were designed to act on their binding region [191]. The Cas9 enzyme can then introduce a variety of different types of mutations to the binding site of the homing guide RNA to act as cellular barcodes. They applied this method to the developing murine brain, assessing barcodes from the left and right sides of the forebrain, midbrain, and hindbrain. Their results found that commitment to the anterior-posterior axis occurs before the lateral axis; however, they did not resolve this pattern in the neocortex itself. The homing CRISPR technique shows the feasibility of utilizing engineered mosaicism to identify and track lineage, both spatially and temporally. While technically different, the use of Cre recombinase on the complex engineered ‘Polylox’ locus follows a similar logic [192]: here, combinatorial recombination distinguishes distinct lineages, as was demonstrated in the hematopoietic system. Common to all these techniques is the ability to retrieve the combinatorial and lineage-defining loci through direct sequencing as the genomic tape is known *a priori*. Following the retrieval of the engineered mosaic marks, specialized algorithms allow the reconstruction of lineages similar to genome-wide natural mosaicism.

The two biggest advantages of using such methods are the tunable resolution of lineages based on the activity of the writer and the reduced sequencing cost due to the known mutated genomic tape. However, these described methods are inherently unable to reflect any ‘cellular states’ other than their initiation condition (e.g., expression of a Cre recombinase). In response to this limitation, several alternative approaches have been developed that allow the recording of such states (Fig. 5C). Fundamentally, these methods aim to reflect features such as gene expression or protein binding to chromatin as a permanent record in the genome.

For instance, a study by Chen and colleagues proposes the driving of multiple writers with distinct signatures by distinct enhancers [193]; this would enable to recording of a temporally resolved sequence of a predetermined number of input signals and use them for lineage tracing. Going one step further, bacterial systems can store copies of expressed RNA in their genome [194–196]. While currently only employed to record highly expressed RNAs, this has the potential to also mark lineages by variable integration of these transcripts. However, these systems have not yet been translated into eukaryotes or employed to distinguish clones of cells. Thus, a genome-wide recorder of transcriptional activity is currently unavailable in mammals. Finally, a method named ‘Calling Cards’, developed by the Mitra laboratory provides a distinct recording of cellular state [197, 198]. This method marks binding sites of transcription factors through the integration of a permanent transposon into the genome. Therefore, the genome-wide binding of a protein of interest can be assessed based on a customizable genomic scar.

While engineered mosaicism has advantages over natural mosaicism in terms of lineage tracing, there are important applications of the latter in model organisms. For instance, a recent study by Uchimura and colleagues tracked natural mosaicism in somatic and germ cell lineages for lineage tracing ^[199]^. Importantly, their approach allowed them to retrieve features of mutation rates and signatures, which allows the inferral of the mutational environment during development. Moreover, the use of natural mosaicism allows the avoidance of potentially complex breeding strategies to introduce the necessary tunable genomic writers and the genomic tape. As sequencing costs continue to decrease, the most important advantage of engineered mosaicism is its superior resolution. However, it is conceivable to modulate mutation rates through the use of chemical mutagens or genetic backgrounds with impaired genome repair mechanisms. While currently not optimized, the bioinformatic analysis could be adapted for model organisms as needed.

## Conclusions

Driven by technological advances, genomic mosaicism research in the brain has progressed significantly in the last decade. This review provides a bird’s eye view of current trends in this field, including conceptual definitions, current methodological approaches, and an overview of the impact and utility of genomic mosaicism. As sequencing is one of the main drivers of current discovery, this field benefits from the rapidly decreasing costs of next-generation and—prospectively—third-generation sequencing. This allows researchers interested in lineage tracing questions in the brain to more easily apply genomic mosaicism approaches. We hope this review will act as a primer for interested parties and allow wider adoption of the here-described concepts.
